# A sampling-based approach for information-theoretic inspection management

**DOI:** 10.1098/rspa.2021.0790

**Published:** 2022-06

**Authors:** Lawrence A. Bull, Nikolaos Dervilis, Keith Worden, Elizabeth J. Cross, Timothy J. Rogers

**Affiliations:** ^1^ Dynamics Research Group, Department of Mechanical Engineering, University of Sheffield, Mappin Street, Sheffield S1 3JD, UK; ^2^ The Alan Turing Institute, The British Library, London NW1 2DB, UK

**Keywords:** Structural Health Monitoring, Active Learning, Semi-Supervised Learning, Dirichlet Process, Generative Mixture-Models

## Abstract

A partially supervised approach to Structural Health Monitoring is proposed, to manage the cost associated with expert inspections and maximize the value of monitoring regimes. Unlike conventional data-driven procedures, the monitoring classifier is learnt online while making predictions—negating the requirement for complete data before a system is in operation (which are rarely available). Most critically, periodic inspections are replaced (or enhanced) by an *automatic* inspection regime, which only queries measurements that appear informative to the evolving model of the damage-sensitive features. The result is a partially supervised Dirichlet process clustering that manages expert inspections online given incremental data. The method is verified on a simulated example and demonstrated on *in situ* bridge monitoring data.

## 1. Introduction

Expert inspections can contribute significantly to the cost of Structural Health Monitoring (SHM) regimes [1]. In practice, the inspection budget is often limited by some expense or difficulty associated with expert elicitation; for example, consider visual inspection of bridges or (manual) non-destructive testing of an offshore wind turbine blade. Despite the associated cost, the information provided by experts proves critical in data-driven monitoring, as it can be used to label (i.e. annotate) the measurements from operational systems. Label information is essential to provide *supervised* data, to learn models that classify the system into a number of operational, environmental or damage states—rather than simply indicating normal or abnormal operation (novelty detection [2]).

In particular, inspection management is critical where monitoring algorithms are learnt online from *data streams*—i.e. measurements that arrive incrementally in (near) real-time [3,4]. Such data are increasingly prevalent in emerging applications of bridge monitoring [5,6], manufacturing [6–8] and wind power [9–11]. Here, the inspections must be managed and integrated, given online changes in the data, especially when excessive labelling fails to increase the understanding of the system condition. A practical example exists in operations and maintenance for offshore wind, where uninformative inspections suggested by data-driven monitoring (because of false negatives) are an acknowledged critical concern [10].

In such scenarios, *active* and *semi-supervised* learning can be used to automatically manage and introduce label information via guided system inspections, while considering the associated *cost* [12]. In general terms, active learning assumes that an algorithm can improve performance, from fewer labels, if it can select the data from which it learns [13]. On the other hand, semi-supervised learning formally combines a small budget of labels (system inspections) with large volumes of unlabelled signals [12].

Semi-supervised methods have brought improvements in various application domains. One example considers protein classification—since protein sequences are complex, it is impractical to analyse every sequence in order to label their type. The work in [14] shows that using the remaining unlabelled sequences (alongside a budget of labelled examples) greatly improves the classification performance. Another classic example considers text-document classification [15], where semi-supervised updates increase the classification accuracy from 59% to 70% for the *20 Newsgroups* benchmark data (using just 20 labelled documents per class). Finally, the results in [16] also present improvements when classifying the MNIST benchmark—achieving a 12% error using just 4% labelling (of 10 000 training images).

### (i) Contribution

This work proposes that system inspections are guided via a constrained Dirichlet Process Gaussian Mixture Model (DPGMM) of data recorded from operational engineering systems. The result is a partially supervised monitoring regime [17], with three key advantages over conventional techniques:

• **The model negates the requirement for large** ****comprehensive**** **data^1^ *a priori***: instead, the model is updated given changes in the incremental data.
• **The value of the monitoring regime is improved via guided inspections**: selecting the most informative observations to inspect, and combining those limited inspections with large volumes of measured data.
• **The number of operational, environmental and health states does not need to be pre-defined**: instead, novel classes of data are included in the model as they are discovered (via expert inspection).

The layout of the paper is as follows. Section 2 discusses the issues of learning from limited expert inspections (and therefore labels) in performance and health monitoring—active and semi-supervised learning are introduced from a probabilistic perspective. Section 3 introduces DPGMMs in the context of monitoring. Sections 5 and 4 modify the Dirichlet process mixture model to incorporate a small budget of expert labels, while §6 verifies the technique on simulated SHM data. Section 7 provides a practical case study (*in situ* bridge monitoring data), and §8 offers some concluding remarks.

## 2. Partially supervised learning for data-driven monitoring

In the ideal scenario, an engineer would know the specific condition of a structure from measured data alone. In practice, inspections must be carried out to reveal the condition, which is economically infeasible for every point in time. In SHM, therefore, related data are used to infer the system condition, often from some sparse set of labels, in conjunction with the patterns observed in previous measurements.

Table 1 provides examples where comprehensive labelling of the operational, performance or health state data via inspection proves problematic. In these scenarios, descriptive labels for each measurement are economically infeasible or impractical. This problem renders many conventional data-driven *classification* models inapplicable in monitoring applications [1,2], as they require fully labelled or *supervised* training data. In other words, each measurement vector $x_{i}\in R^{d}$ must have its own descriptive (scalar) label $y_{i}\in R$ to define a supervised training-set,

$$D_{l}={\left\{{\text{x}}_{i},y_{i}\right\}}_{i=1}^{N},$$

where N is the number of training samples used to *learn* the monitoring classifier $f(x_{i})=y_{i}$ and $y_{i}\in \{1,\ldots ,K\}$ is a descriptive label for point i. The label associates the structure with one of K operational, environmental or health conditions.

**Table 1.** Factors limiting the inspection budget.

Label (inspection) limitation	Example
There is some high cost associated with the data labelling process itself	Expert inspection via non-destructive testing or evaluation procedures [19]
It is infeasible to inspect the structure because of accessibility issues	Offshore wind farms [9,10]
Some high cost is associated with downtime	Suspending machining operations to inspect the cutting tool in a lathe during high-value manufacturing [7,8]
Data annotation is informed by some physics-based model, which is computationally expensive	Running a finite-element model for a range of possible environmental conditions to label *in situ* measurements [20]

A problem with supervised learning is that, while it allows online assessment, the learner fails to make use of the available unlabelled data seen during the evaluation stage, i.e. while a structure is in operation. That is, the full value of the monitoring system is not realized, as those in-service measurements—that remain unlabelled—are discarded after being evaluated online by the classifier.

Conventionally [21], the absence of labels ${\left\{y_{i}\right\}}_{i=1}^{N}$ in SHM will force a dependence on *unsupervised* learning: data-driven models learnt from measurements ${\tilde{x}}_{i}$ only. As such, the training data are

$$D_{u}={\left\{{\tilde{\text{x}}}_{i}\right\}}_{i=1}^{M},$$

where M is the number of unlabelled training samples. The choice to adopt unsupervised methods is generally related to the cost, both financial and in terms of time, to acquire the labelling ${\left\{{\tilde{y}}_{i}\right\}}_{i=1}^{M}$. While unsupervised techniques have proved successful in many applications [2,21] they limit monitoring procedures to *novelty detection* [22]; i.e. an indication of *normal*, or *abnormal* operation only. This limitation can be clarified under Rytter’s hierarchy [2,23] for damage detection:

(i) *Detection*: an indication that damage might be present.
(ii) *Location*: an estimate of the location of damage.
(iii) *Classification*: a prediction of the type of damage.
(iv) *Assessment*: an estimate of the extent of damage.
(v) *Prediction*: a method for prognosis.

In general terms, each level increases in difficulty and this requires more information for reliable predictions. Without label information or supervision (in some form), it is widely acknowledged that progressing up the hierarchy proves increasingly difficult and mostly infeasible in practice [1].

However, in many applications it is feasible to label a *small* number of measurements, given a budget determined by the performance/health monitoring regime, for example:

• The cutting tool in a lathe could be inspected between turning operations,
• or a wind turbine could be inspected during scheduled maintenance trips.

In these scenarios, there are two sets of training data to consider: the supervised set $D_{l}$ and the unsupervised set $D_{u}$. Because of budget^2^ restrictions, the number of labelled data will generally be much smaller than the number of unlabelled data; i.e. M≫N. Considering the large volumes of data that remain unlabelled, the associated information might be used to build a better understanding of the behaviour of the structure (alongside labelled data).

More precisely, with both labelled and unlabelled data, it would be limiting to learn a classification algorithm given only $D_{l}$ while ignoring information in $D_{u}$; likewise, the converse is true—provided that information can be extracted and combined in a meaningful manner. Instead, data-driven modelling should use the labelled and unlabelled data in a *combined* approach, such that the union set is considered,

$$D=D_{u}\cup D_{l}.$$

Conveniently, there are statistical and machine learning tools designed for learning from partly labelled data; these are referred to as *partially supervised* algorithms [12]—this is used here as an umbrella term, to refer to methods of *learning from fewer labelled examples*.^3^

In the suggested framework two different forms of partially supervised learning are exploited in the context of data-driven monitoring; namely, *semi-supervised* and *active* learning. The details of which are now considered in more detail.

### (a) Deciding when to label: probabilistic active learning

For active learners, the main premise is to improve the predictive performance of the mapping $f(x_{i})=y_{i}$ as far as possible while requesting (querying) a limited number of labels [13]. In most scenarios, queries are taken from the unlabelled data in $D_{u}$ to automatically extend the labelled data $D_{l}$.

Generally, there are two main settings for active learning: *stream-based* and *pool-based* [13]. In stream-based methods, the data in $D_{u}$ arrive incrementally (in real-time) and the active learner must determine whether to query, or not, at that instance. The learner cannot obtain a label for any data other than the most recent measurement. On the other hand, pool-based methods iteratively select the most informative datum from a static set of unlabelled examples—here, the label of any datum can be investigated at any time. Intuitively, active learning has the potential to assist inspection management in SHM as the learner can automatically suggest measurements for which inspections appear necessary to improve (or maintain) the predictive performance of f. A critical step, therefore, is determining which data should be investigated and labelled.

It is worth noting that, in almost all SHM settings, the active learning problem will be stream-based, since it is generally not possible to look at the condition of the structure backwards in time; instead, only the current condition may be investigated. For this reason, the focus of this work is on developing a stream-based active learner for SHM.

Perhaps the most obvious way to query data is to select instances that appear uncertain, given the current model [6]—this procedure is known as *uncertainty sampling* [13]. Starting from a small number of labelled data, further points are queried according to those that appear ‘uncertain’ based on various statistics. For example, entropy can be used to query observations whose *predicted labels* appear to be the most ‘confused’ or ‘conflicted’, referred to as Maximum Entropy Sampling (MES) [25]. Typically, the Shannon entropy [26] of the posterior-predictive-distribution over the unobserved labels $p({\tilde{y}}_{i}|{\tilde{x}}_{i},D_{l})$ is used,^4^

2.1 $$H({\tilde{y}}_{i})=-\sum_{k=1}^{K}p({\tilde{y}}_{i}=k|{\tilde{x}}_{i},D_{l})\log p({\tilde{y}}_{i}=k|{\tilde{x}}_{i},D_{l}).$$

The result of querying labels with maximum entropy is to select those data that appear at the boundaries between existing classes (i.e. data which could be explained by one or more structural conditions). For example, SHM measurements whose classification into a normal condition or an environmental effect is equally likely—these data will lie on the boundary between those two classes and will also lie at the point of maximum entropy.

Another view of uncertainty sampling considers data ${\tilde{x}}_{i}$ that appear *unlikely*, given the current model. In contrast to MES, low-likelihood samples [6] query data that appear at the extremities of the model, rather than at class boundaries—i.e. SHM data which the model cannot approximate with any known structural condition. In terms of probability distributions, low-likelihood measurements can select those with a low (marginal) likelihood

2.2 $$p({\tilde{\text{x}}}_{i}|D_{l})=\sum_{k=1}^{K}p(\tilde{\text{x}}|{\tilde{y}}_{i}=k,D_{l})\;p({\tilde{y}}_{i}=k|D_{l}).$$

These queries are useful in discovering new classes of data as they sample measurements that appear *novel* given the model (rather than confused). Such queries are arguably most useful in novelty detection—when applying active learning to streaming data, for example [6].

Another view considers a Bayesian experimental design perspective [27]. In words, select data that appear to *improve the model* as quickly as possible. The work in [28] proposes a querying scheme by selecting observations whose labels are expected to lead to the greatest reduction in entropy of the posterior distribution over the parameters of some Bayesian classifier, herein denoted generally as θ. That is, labels that provide the most information about the model (via θ) when queried. This is typically formalized by defining a *utility* for querying the point ${\tilde{x}}_{i}$,

2.3 $$U({\tilde{x}}_{i})=H(p(\theta |D_{l}))-E_{p({\tilde{y}}_{i}|{\tilde{x}}_{i},D_{l})}[H(p(\theta |D,\{{\tilde{x}}_{i},{\tilde{y}}_{i}\}))].$$

As with MES, the entropy here is the Shannon entropy—which is used to quantify model uncertainty via some probability distribution. However, unlike expression (2.1), this utility is concerned with the entropy of the posterior distribution over the parameters p(θ|D)—rather than the predictive distribution over the labels $p({\tilde{y}}_{i}|{\tilde{x}}_{i},D_{l})$. A reduction in the entropy of the distribution of the parameter estimates implies a reduction in the uncertainty of the model.

### (b) Combining labelled and unlabelled data: semi-supervised learning

Another partially supervised technique suited to performance/health monitoring is *semi-supervised* learning [12,29]. The focus here is to use the remaining unlabelled data in $D_{u}$ to help infer the parameters of the classifier. Therefore, the model $f(x_{i})$ is now learnt from the union-set of labelled and unlabelled data $D=\{D_{u}\cup D_{l}\}$ within a *unifying* training scheme.^5^

Semi-supervised models have potential in performance/health monitoring as a small set of labels (provided by the engineer) can be combined with the larger sets of unlabelled measurements. Unsurprisingly, there are numerous ways to enforce semi-supervision. Arguably, the most interpretable is *self-training* (also self-labelling, pseudo-labelling) [24,29]. In simple terms, the predicted labels for ${\tilde{x}}_{i}$ are used as *pseudo-labels* to train the algorithm in subsequent learning steps.

Returning to themes of entropy, self-labelling implicitly encourages models with low-entropy predictions (i.e. confident label predictions) [24]. Formally, *entropy minimization* techniques [30] can be viewed as minimizing the following loss function for the unlabelled data [24],

2.4 $$L=-\sum_{i=1}^{M}\sum_{k=1}^{K}p({\tilde{y}}_{i}=k|{\tilde{x}}_{i})\log p({\tilde{y}}_{i}=k|{\tilde{x}}_{i}).$$

One notices similarities to the entropy expression for active learning (2.1) and that (2.4) is minimized when points are assigned to a single class of data with unit probability. In simple terms, the parameters of the model θ are adjusted such that the unlabelled data are classified with the maximum-possible distinction.

A special case can be implemented for generative mixture models [31] via Expectation Maximization (EM), originally proposed by Almeida *et al*. [15], such that the expected joint log-likelihood is maximized [29],

2.5 $$\begin{array}{rl} L(\theta |D) & \;=L(\theta |D_{u},D_{l})\propto \underset{D_{u}}{\underbrace{\sum_{i=1}^{M}\log\sum_{k=1}^{K}p({\tilde{x}}_{i}|{\tilde{y}}_{i}=k,\theta )p({\tilde{y}}_{i}=k|\theta )}}\ldots \\ & \;\;+\underset{D_{l}}{\underbrace{\sum_{i=1}^{N}\log[p({\text{x}}_{i}|y_{i}=k,\theta )p(y_{i}=k|\theta )]}}+\log p(\theta ). \end{array}$$

Expression (2.5) implies that the full joint log-likelihood of the model is maximized, considering *both* the labelled data (term one) and unlabelled data (term two). For details of how (2.5) relates to an entropy minimization viewpoint, refer to [30,32].

### (c) Semi-supervised and active monitoring

These motivations of partially supervised learning directly align with the goals of data-driven monitoring in an engineering setting. The similarities have been recognized and recent advances in the literature demonstrate either active or semi-supervised approaches to health monitoring; a brief review is provided.

#### (i) Semi-supervised learning

In bridge monitoring applications, Chen *et al.* introduced a graph-based approach for label propagation [33,34]. The objective function of a multi-resolution classifier [35,36] is modified such that the weighting parameters are optimized over the labelled and the unlabelled data. Another graph-based algorithm is applied for fault diagnosis in condition monitoring of bearings and pumps [37]. Label propagation within hierarchical clustering has been investigated with experimental aircraft data [38] and pipe monitoring [39]. Generative mixture models have also been adapted for probabilistic and semi-supervised monitoring with vibration-based aircraft data [40].

Further methodologies that are considered related to semi-supervised SHM consider applications of K-means [41], Dirichlet Processes and fuzzy-C-means [42] clustering. Huang *et al.* [42] use fuzzy-C-means within an online SHM strategy; the proposed method becomes partially supervised during a *label-matching step*, where the unsupervised clusters are compared with known classes from the supervised data. Bouzenad *et al.* [41] define a similar online framework using K-means where new clusters are created when a distance-based threshold is crossed within the unsupervised algorithm. In these examples, partial supervision is enforced within the SHM framework, rather than the inference.

#### (ii) Active learning

Active learning is somewhat less explored in performance and health monitoring applications. Existing studies include generative mixture models for clustering of data in tool-wear [43] and bridge monitoring regimes for information-theoretic [6] and decision-theoretic [44] procedures. Neural networks have been applied with uncertainty sampling to classify images of defects in a dataset concerning civil structures [45]. The work [46] proposes a Bayesian convolutional neural network for tool monitoring, using MES. Finally, an adaptive probabilistic framework is proposed in [47] for active data selection to aid a particle filter-based damage-progression model.

#### (iii) A new view of partially supervised SHM

The overarching aim of the existing literature is to either: (i) make use of all the measured data (semi-supervised learning), *or* (ii) determine which measurements require inspection (active learning). While these are the goals of the methodology proposed here, the benefits from both active and semi-supervised learning will be *combined* in a single algorithm. Furthermore, the ambition is extended, to automatically suggest an appropriate query budget given the application.

## 3. Monitoring with Dirichlet process mixture models

Having reviewed the relevant forms of partially supervised learning, the underlying probabilistic model, which forms the basis of the proposed approach, is introduced.

### (a) A visual dataset

A simulated (vibration-based) dataset is considered for demonstration and benchmarking. The data are based on an eight-degree-of-freedom system designed by the Los Alamos National Laboratory for SHM purposes [2]. Following identification of the system parameters via modal analysis, time-series data were simulated for six conditions. Each health-state represents progressive damage, approximated via reductions in the stiffness of the system (a common assumption in the literature [2,22]). These reductions take place at spring $k_{5}$, which is located between mass four and mass five, as was the case in the laboratory study:

• $\left[y_{i}=1\right]$ normal (system parameters unchanged),
• $\left[y_{i}=2\right]$ damage 1: stiffness 97%,
• $\left[y_{i}=3\right]$ damage 2: stiffness 93%,
• $\left[y_{i}=4\right]$ damage 3: stiffness 88%,
• $\left[y_{i}=5\right]$ damage 4: stiffness 82%,
• $\left[y_{i}=6\right]$ damage 5: stiffness 70%.

**Figure 1.**
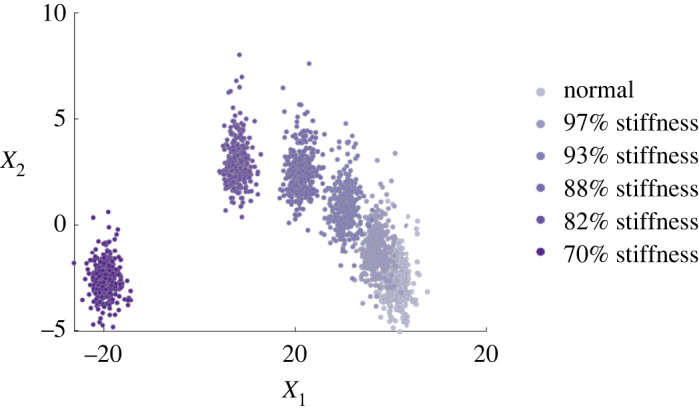
Simulated training data representing progressive damage: increasing severity shown by darker purple clusters. Plotted are the first two principal components of the transmissibility from mass eight to mass one, see [40]. (Online version in colour.)

From the simulated time series, 8-s windows were converted to the frequency domain (transmissibilities) to define 500 frequency-domain observations per class (3000 in total), according to the procedure in [40]. Of these data, 2010 points are set aside for training ($D=D_{l}\cup D_{u}$) and 990 are held out as an independent test-set $D^{*}$. The frequency-domain features are projected via principal component analysis onto two dimensions to visualize the data and fit the model—the principal component subspace is shown in figure 1. At lower damage levels, there is increased confusion/mixing between clusters to define a challenging feature-space.

### (b) Infinite Gaussian mixture models

The conventional DPGMM is an unsupervised Bayesian algorithm for non-parametric clustering. The model can be used to perform *online* inference for SHM, such that the need for extensive training data (before practical monitoring) is mitigated [48]. Bayesian properties of the Dirichlet Process (DP) allow the incorporation of prior knowledge and updates of belief, given the observed data. Prior distributions can help mitigate the need for comprehensive labelling, as the available information can be formally included as prior knowledge. There are relatively few user-tuned parameters, so DP clustering can be implemented to perform powerful online learning with reduced engineering input/knowledge (i.e. access to data or a physical model [48]).

A popular analogy to describe the DP (for clustering) considers a restaurant with an infinite number of tables [49]; i.e. tables represent clusters, labelled by the scalar $y_{i}\in Z^{+}$. Customers, resembling observations $x_{i}$, arrive and sit at one of the tables (according to some probability), which is either occupied or vacant. As more people sit at a given table, the probability that a customer knows someone, and joins the table for dinner, increases. The resulting seating arrangement can be viewed to represent a DP mixture.

For SHM, online clustering via the DP can negate the requirement for the operator to specify an expected number of normal, environmental or damage conditions (i.e. K), prior to learning the model—this can be difficult, or impossible, to define for a structure in practice [48]. Instead, an appropriate number of components is automatically determined by the model, detailed below.

### (c) Model definition

Formally, in a DPGMM, each component in the mixture is described by an independent Gaussian distribution. That is, observations $x_{i}$ (conditioned on the class component $y_{i}$) are Gaussian distributed with mean $\mu_{y_{i}}$ and covariance $\Sigma_{y_{i}}$. In a Bayesian manner, conjugate priors are placed over $\mu_{y_{i}}$ and $\Sigma_{y_{i}}$. Here, the prior over the mean values is a multivariate Gaussian (N), and the prior over the covariance matrices is Inverse Wishart (IW),

3.1*a* $$\begin{array}{r} {\text{x}}_{i}|y_{i}∼N({\text{x}}_{i}|\mu_{y_{i}},\Sigma_{y_{i}}) \end{array}$$

3.1*b* $$\begin{array}{r} \mu_{y_{i}}|\Sigma_{y_{i}},y_{i}∼N\left(\mu_{y_{i}}|\mu_{0},\frac{\Sigma_{y_{i}}}{\kappa_{0}}\right) \end{array}$$

3.1*c* $$\begin{array}{r} \Sigma_{y_{i}}|y_{i}∼IW(\Sigma_{y_{i}}|\Sigma_{0},\nu_{0}) \end{array}$$

with prior parameters $\mu_{0},\kappa_{0},\Sigma_{0}$ and $\nu_{0}$. A multinomial distribution is placed over $y_{i}$, which defines the likelihood of the data point being drawn from a class component 1 to K, with the mixing proportions defined by π. In this case, an appropriate conjugate prior for the multinomial is the Dirichlet distribution, governed by the *dispersion value* α,

3.2*a* $$y_{i}|\pi ∼\text{Mult}(\pi )$$

3.2*b* π∼Dir(α)

**Figure 2.**
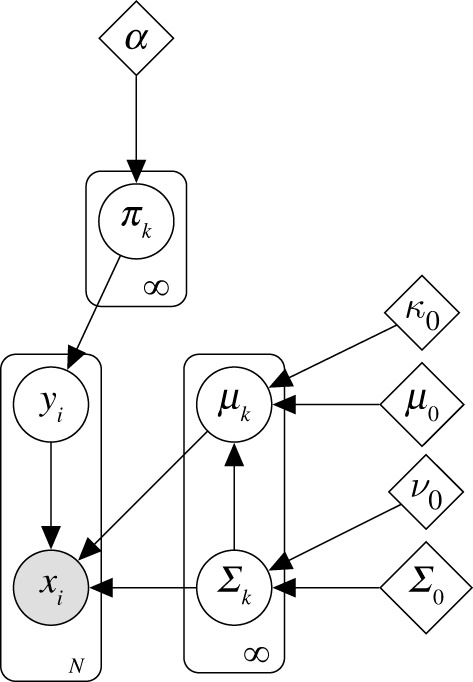
Graphical model of the infinite Gaussian mixture model.

Following [50], it is possible to take the limit K→∞ and form an infinite Gaussian mixture model, for which the generative equations are (3.1) and (3.2); the corresponding graphical model is in figure 2. Importantly, the hyperparameter α encodes the likelihood that data form a new cluster (or table, in the analogy) over an existing one. Thus, α is sometimes referred to as the *dispersion value*, as high values lead to an increased probability that new clusters are formed while low values lead to less. This effect highlights the useful property of DP mixtures: the number of clusters K (i.e. tables) does not need to be defined in advance; instead, this is determined by the model and the data (as well as α) [51]. As a result, the DP can cluster SHM signals online, as the model can adapt and update—selecting the most appropriate value for K as new information becomes available.

A collapsed Gibbs sampler can be used to perform efficient online inference over this model [52]. Within the Gibbs sampler only components k={1,…,K+1} need to be considered to cover the full set of possible clusters [53], since the formulation in [49] represents the infinite set {K+1,…,∞} with the prior distribution. There are two conjugate pairs in the model; therefore, the predictive equations remain analytical (leading to a *collapsed* Gibbs sampler). In brief/general terms: while fixing the parameters, the Gibbs scheme determines the likelihood of an observation $x_{i}$ being sampled from an existing cluster k={1,…,K}, or an (as yet) unobserved cluster k=K+1 (i.e. the prior). Given the posterior over the K+1 classes, the cluster assignment ${\tilde{y}}_{i}$ is sampled, and the model parameters are updated accordingly. This process is iterated until convergence.

## 4. A semi-supervised Dirichlet process

To include inspection knowledge as labels ${\left\{y_{j}\right\}}_{j=1}^{N}$ the DPGMM is modified to learn the parameters in a joint inference over two sets of data:

• An *unsupervised* set, with unknown labels,
• A supervised set, for which the engineer (or expert) has provided *user labels*, linking the data to some human interpretation.^6^

**Figure 3.**
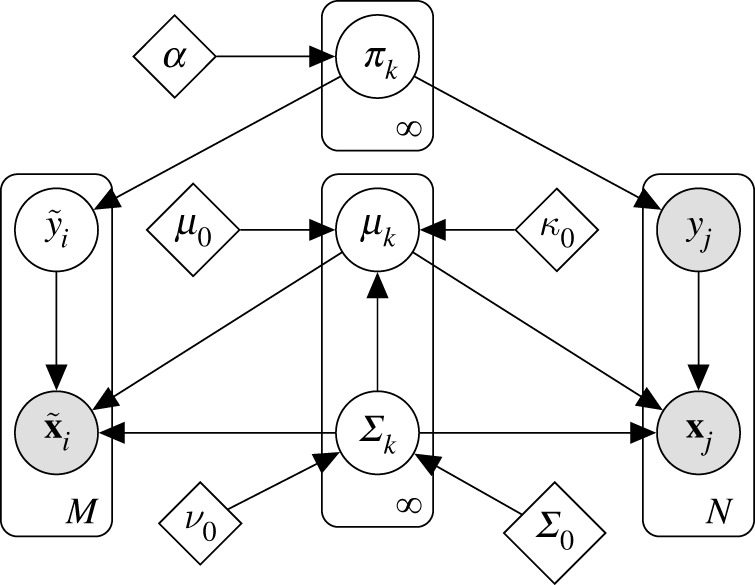
Graphical model of the semi-supervised Dirichlet process Gaussian mixture model.

User labels do not necessarily require a physical or manual inspection; instead, they require some form of (costly) expert insight—for example, analysis of the measured data or the environment. Continuing notation, the model considers N labelled data $D_{l}={\left\{x_{j},y_{j}\right\}}_{j=1}^{N}$ and M unlabelled data $D_{u}={\left\{{\tilde{x}}_{i}\right\}}_{i=1}^{M}$, which share the same generating distribution for $\{x_{i},{\tilde{x}}_{i}$}; thus, the combined training data become $D=D_{l}\cup D_{u}$. The semi-supervised DPGMM is represented graphically in figure 3.

For the supervised data, expert inspections effectively enforce a set of *must-link* constraints on the algorithm: if two points $\left(x_{i},x_{j}\right)$ are observed with the same *user label* they must be sampled from the same underlying Gaussian, implying $y_{i}=y_{j}$. As inference is performed over the model shown in figure 3 by means of the collapsed Gibbs sampler [52], it is necessary to modify this procedure to account for labelled instances. The proposed implementation adopts a similar strategy to Vlachos *et al.* [51], modifying the approximate inference to enforce constraints on the algorithm.

Inspected measurements are assigned a user label c linked to a cluster index k by virtue of an injective map; i.e. $k_{1}\neq k_{2}\Rightarrow c_{1}\neq c_{2}$, where $k_{i}$ is the cluster label of point i and $c_{i}$ the user label of point i. For any data with the user label c the label distribution^7^ $p(y_{j}|D_{u},D_{l,-j})$ is considered to be a multinomial, with all probability centred on the associated cluster index $y_{j}=k$ and a zero-likelihood that $y_{j}\neq k$ (via the map). The distribution is fixed within the Gibbs sampler such that all data annotated with c share the same index k. In effect, this is a *must-link* constraint between observations with the same user description. In turn, the model assumes that each class of data k={1,…,K} is approximated by a single Gaussian component.

To demonstrate semi-supervised improvements to the DPGMM, it is applied to the simulated data. The hyperparameters are set consistently throughout this work: the prior mean is the expected value of the data $\mu_{0}=E[x_{i}]$ and the prior covariance is based on the (expected) variance $\Sigma_{0}=(\nu +d+2)\times V[x_{i}]$—determined empirically or by prior intuition; following standard practice in monitoring applications [6,40,48]: κ=1, ν=d and α=10.

**Figure 4.**
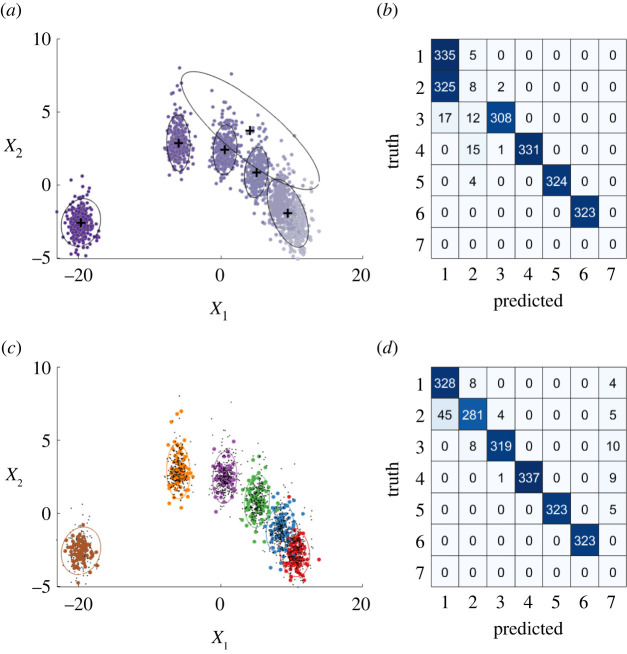
Model improvements via semi-supervised updates to the DPGMM. (a, b) The unsupervised DPGMM, colours and features as in figure 1. (c,d) A semi-supervised DPGMM with 26.8% labelling—this budget, in effect, leads to a 73.2% reduction in labelling cost compared with the fully supervised model. (a, c) Diagrammatic representations of the clustering procedures, noting that labels must be assigned manually in the unsupervised case. (b, d) Confusion matrices where the label switching problem is manually fixed in the unsupervised case. (Online version in colour.)

Figure 4 shows significant improvements over the unsupervised case when including a subset of 26.8% labels (536 points). Confusion matrices are also shown in figure 4 to highlight the improvement in the performance of the semi-supervised model. It should be noted that, in the unsupervised case, labels are not available to link the clusters to structural conditions. For comparison, the unsupervised clusters have been manually labelled by the authors—even when including this manual intervention, it can be seen that the unsupervised model is insensitive to the change between classes one and two, whereas the semi-supervised learner is. Quantitatively, the test accuracy increases from 80.9% to 96.2% when labelling is used to constrain the DP.^8^

## 5. Guided inspections: a ‘probability-of-query’ sample scheme

The active sampling scheme is based on the entropy of the posterior distribution of the label ${\tilde{y}}_{i}$ at a given step in the Gibbs sampler,

$$H\{p({\tilde{y}}_{i}=k|{\tilde{x}}_{i},D_{-j})\}=-\sum_{k=1}^{K+1}p({\tilde{y}}_{i}=k|{\tilde{x}}_{i},D_{-j})\log\{p({\tilde{y}}_{i}=k|{\tilde{x}}_{i},D_{-j})\}.$$

This expression can be interpreted as the uncertainty in classifying a measurement as a previously observed structural condition, or an (as yet) unobserved condition, encoded in the K+1 prior.

The information efficiency [26] is then defined, giving rise to a normalized value between zero and one,

5.1 $$\eta ({\tilde{x}}_{i})=\frac{H\{p({\tilde{y}}_{i}|{\tilde{x}}_{i},D_{-j})\}}{\log(K+1)}.$$

Here, (5.1) is viewed as an approximation of how likely observing the ground truth of ${\tilde{y}}_{i}$ will improve the model. It represents the *confidence* in the label prediction, compared with the assumption that all conditions are equally likely.

$\eta ({\tilde{x}}_{i})$ can then be treated as a *pseudo-probability* that datum ${\tilde{x}}_{i}$ should be queried—the *probability of query*. Specifically, labels are queried if a sample from a random variable a∼U(0,1) is less than $\eta (x_{i})$. The resulting queries favour points with a high entropy in the label posterior over the infinite mixture model. Although similar to conventional MES active learning (2.1), by considering the K+1 cluster (i.e. the prior) points at the extremities of the model can be sampled as well as points at the class boundaries. The reason for sampling at the extremities is the confusion between the classes containing data and the prior (which contains no data). In turn, the active learner combines MES (2.1) and low-likelihood (2.2) sampling behaviour—two types of uncertain data that are expected in SHM applications. The benefit is that varied queries should naturally protect against sampling bias [13]: an effect whereby observations of a certain type are labelled too frequently (leading to unrepresentative data). The protection against sampling bias should be enhanced by the probabilistic sampling of when to investigate a point since there is always a non-zero probability that a given point will be investigated.

Surprisingly, the introduction of the semi-supervised and active approach serves to lower the computational burden of the clustering procedure. Two additional operations are introduced into the learning process: (i) determining the *probability of query*, and (ii) deciding if a query should be made. The first operation (i) requires computation of equation (5.1)—the complexity here is dominated by the computation of $p({\tilde{y}}_{i}|{\tilde{x}}_{i},D_{-j})$, however, this is already computed as part of the unsupervised DPGMM. The remaining computation of equation (5.1) is negligible. Determining whether to sample (ii) also has very little impact on the computational cost. An overall reduction in the computational load arises from the Gibbs sampler set-up since the investigated (labelled) points do not need to be re-evaluated in the sampler (see figure 3, $y_{j}$ is observed). These fixed points reduce the computational effort of each pass through the data by the Gibbs sampler, as compared with the unsupervised model. Since this operation would be repeated multiple times per data point, the overall computational saving outweighs the additional work of the active learning scheme.

**Figure 5.**
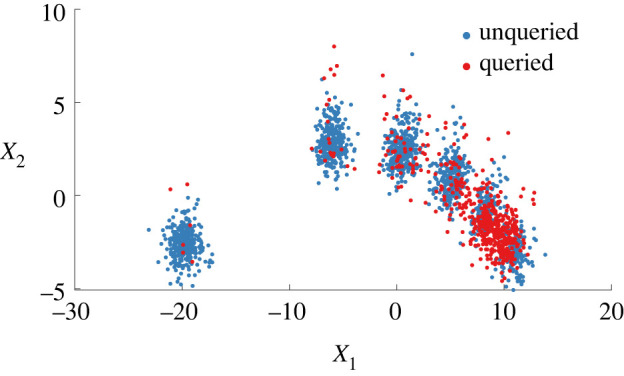
Active queries using the information efficiency as the probability of inspection are shown on the first two principal components of the transmissibility from mass eight to mass one. (Online version in colour.)

To demonstrate, queries from the simulated data are shown in figure 5. Of the 26.8% labels, both novel data (at the extremities) and confused data (at the boundaries) are selected by the learner. Intuitively, mixed clusters are queried frequently while the separable clusters are queried less.

## 6. Offline verification: simulated data

**Figure 6.**
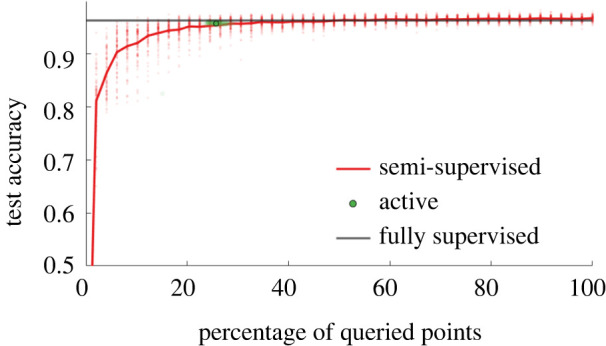
Offline verification: semi-supervised performance for an increasing label budget, the automatic budget selected by the active learner is shown in green. (Online version in colour.)

Initially, the method is verified offline with the simulated data. (It is later applied *online* to the simulated data as well as *in situ* bridge monitoring data.) In a typical manner [29], the proportion of labelled data is increased from 0% to 100% while the classification test accuracy is recorded (averaged over 100 repeats). The corresponding curve is plotted in figure 6—the automatic query budget selected by the active learner (outlined in §5) is plotted in green.

Importantly, figure 6 shows that the active learner selects an appropriate compromise between classification performance and excessive labelling. The algorithm naturally samples a label proportion around the ‘elbow’ of the semi-supervised performance curve—where improvements in the classification performance become less significant with more queries. In practice, it is hard to determine the true *optimal* budget, since there will always be a trade-off between the cost of labelling and predictive performance. However, in this case, the automatic label budget achieves near fully supervised performance (active—96.2% versus supervised—96.3% average accuracy) while using a fraction of the total labels (25.7%). This automatic budget corresponds to 516.3 labels on average (over 100 repeats); in practice, this is a whole number of samples for a given run of the algorithm.

**Figure 7.**
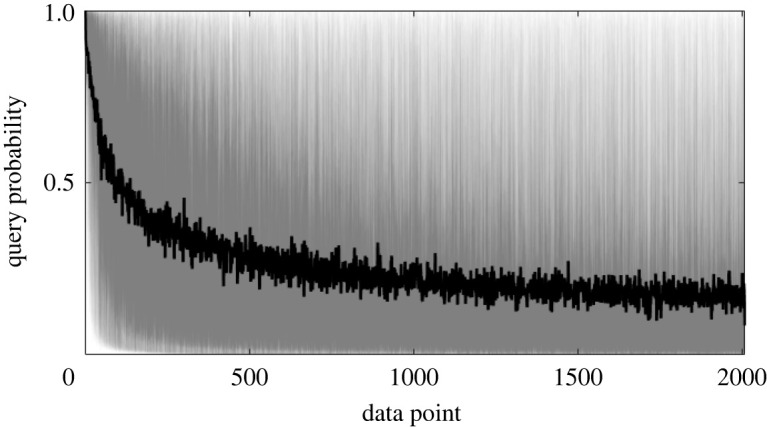
Query probability throughout offline training. The thick black line indicates the mean query probability for the data point seen, the order of data is randomized between repeats, hence the high variance.

Figure 7 considers the (pseudo) probability-of-inspection $\eta ({\tilde{x}}_{i})$ throughout offline training (i.e. randomized data)—this has an intuitive interpretation. The likelihood of inspection is initially high (near unity) because the data are sparse and the underlying density is poorly described. As more data and labels are observed the model becomes increasingly confident that the information sufficiently describes the underlying density; in turn, the probability of inspection gradually falls towards some equilibrium at a low query probability. High variance is seen in the query probability since the offline data are randomized (or shuffled) between repeats, however, the mean trend clearly decreases.

### (a) Towards adaptive clustering of online SHM data

While offline experiments verify the partially supervised DP, SHM applications benefit from models that can be learnt incrementally as data arrive online throughout operation. For this type of inference, the training data are no longer randomized; instead, they arrive ordered, as damage progresses and operational/environmental conditions are introduced.

**Figure 8.**
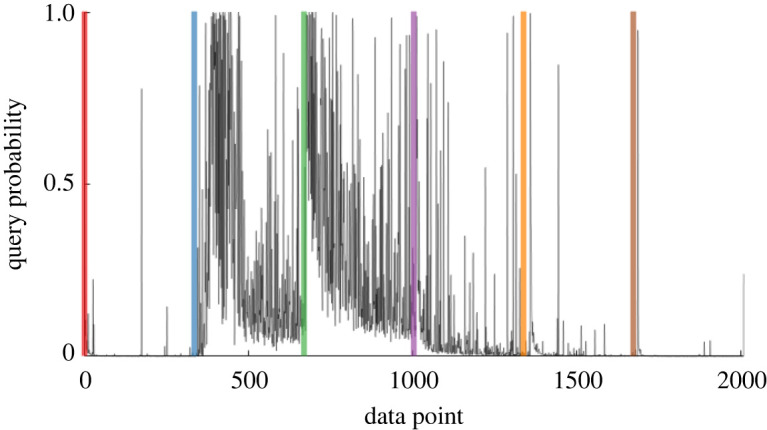
Query probability for the online (ordered) data. Vertical coloured lines indicate the initiation of each cluster. (Online version in colour.)

Figure 8 shows how the proposed methodology handles such incremental data, plotting the probability of inspection for the *ordered* simulated data. Vertical lines highlight the introduction of novel damage conditions throughout training (the colour scheme is consistent). Because of the consistent ordering of the data, a lower variance in the query probability per data point is observed.

As with the offline case, the probability of inspection is near-unity at the start of training. As more data arrive, only a single label is observed (red) and the query probability drops: the model assumes there is enough information to approximate a single class of data. When a new class is introduced (blue) the query probability returns sharply towards near-unity, as the current model poorly approximates the novel data. In turn, labels are queried to discover the new condition and thus reduce the uncertainty in the model. This pattern repeats for each damage condition (corresponding to vertical lines), with the inspection probability falling less dramatically for those classes that are more mixed (blue and green)—this makes sense, as the model remains uncertain of those predictions.

**Figure 9.**
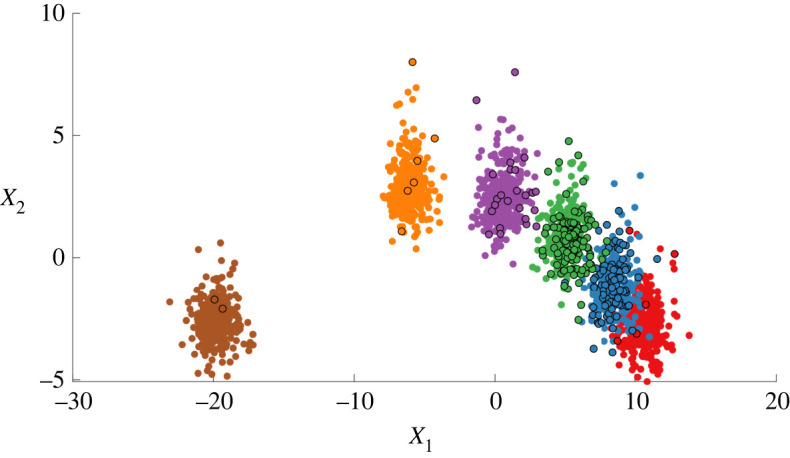
Active queries using information efficiency for online (ordered) training data, the first two principal components of the transmissibility from mass eight to mass one. Queried points are shown by circling the datapoint in black, the colours follow those of figure 8. (Online version in colour.)

To visualize how specific queries adapt to the online case, figure 9 shows the clusters, introduced incrementally from right to left, and the associated inspections (circled). In line with figure 9, the first cluster is queried less, with more samples at the leading edge of newly introduced classes. Likewise, as clusters become more separable, the learner requests fewer labels, as there is less confusion in the predictive entropy of those base distributions.

By comparing figures 9 and 5, it can be seen that far fewer points are investigated by the active learner in the online case than the offline case. Quantitatively, the reduction is almost half with 516 points queried on average in the unordered case compared with 242 in the ordered case. Since the data arrive in order, the model is able to establish the shape of each cluster with fewer labelled points, reducing its variance and leading to decreased confusion between classes. This is important in SHM, as a structure will often remain in a single class for an extended period of time, e.g. the normal condition, which (from this simple illustrative example) may lead to a further reduction in the number of necessary investigations.

## 7. Z24 bridge benchmark data

The DPGMM is now applied to *in situ* measurements from the ‘SIMCES bridge monitoring campaign’ [54]. The project monitored a concrete highway bridge in Switzerland over a 12-month period, before its demolition in 1998. This analysis considers the first four natural frequencies of the structure over time, extracted from data recorded from a series of sensors used to capture dynamic response. Air/deck temperature, humidity and wind speed were also recorded. A total of 3932 observations are in this version of the dataset [55]: critically, before its demolition, damage was artificially introduced into the structure, starting from observation 3476 [56].

In the analysis, the first four natural frequencies are the observations, so that $x_{i}\in R^{4}$. The damage data are assumed to represent their own class, from observation 3476. Outlying observations in the remaining data are determined according to Ranzani *et al*. [6] using the robust Minimum Covariance Determinant (MCD) algorithm [57]. It is believed that these outliers result from the asphalt layer in the deck experiencing very low temperatures during winter—leading to increased structural stiffness (i.e. an environmental condition) [56].

**Figure 10.**
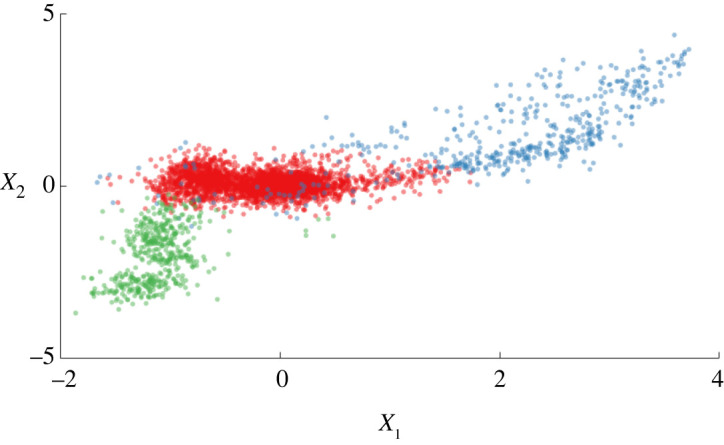
Training data for the Z24 case study: the first two features of the four-dimensional feature space are plotted for visualization. (Online version in colour.)

In line with previous work [6,56], these labels are assumed to represent the ground truth, defining a three-class classification problem (K=3). Figure 10 plots the first two features of the $R^{4}$ feature space: normal data are red, outlying data (due to environmental effects) are blue and damage data are green.

### (a) Why manage labels?

**Figure 11.**
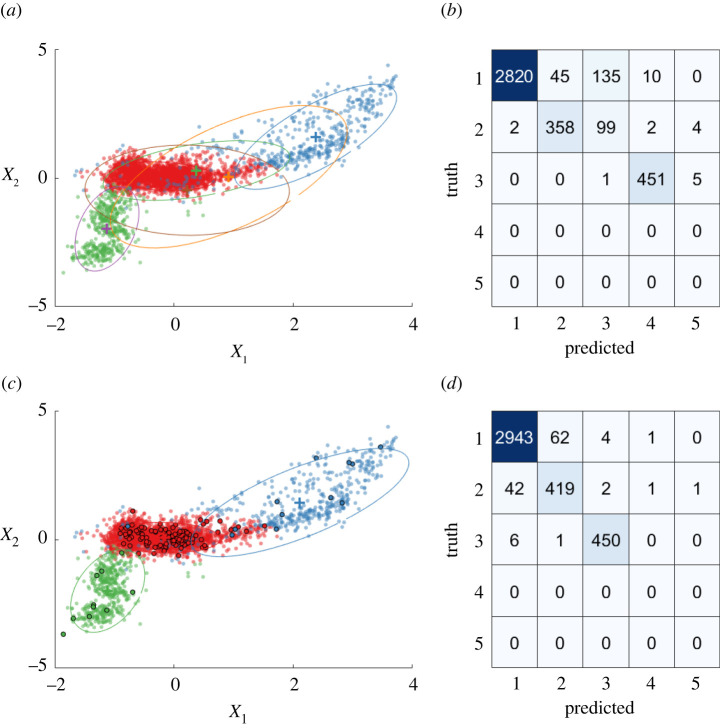
Comparisons between unsupervised and active mixture models for the Z24 data, plotting the first two features of the four-dimensional feature space. (a,b) Unsupervised Gaussian mixture model. (c,d) Active DPGMM. Queried points are shown by a black circle around the marker. (a,c) Plots showing learnt clusters from each model. (b,d) Confusion matrices indicating the assigned labels. (Online version in colour.)

It is impractical for an engineer to annotate each measurement from every data acquisition. For this version of the data, such a fully supervised regime would require ≈ 11 inspections per day. The associated (fully supervised) mixture model achieves 97.20% classification accuracy, which although very accurate, is impractical for applications to an engineering asset. On the other hand, if labels are ignored, the unsupervised mixture model has no formal way to assign user labels to clusters and the unconstrained clustering leads to an inappropriate number of components—for example, the top left of figure 11, where K=6. Poor categorization into meaningful labels is shown by the confusion matrix in the top right of figure 11.

Considering the inadequacies of conventional supervised/unsupervised learning, the partially supervised DPGMM is applied to achieve a trade-off between fully supervised performance and the *cost* of labels. The improvement in performance, as compared with the unsupervised case, is shown at the bottom of figure 11. Labelling a small fraction of points indicated by the active learner, the confusion matrix (bottom right) shows that mislabelling is greatly reduced. Additionally, the algorithm can formally associate user descriptions to clusters of data, while using an automated budget for expert insight to constrain the model.

### (b) Partially supervised SHM with the DPGMM

**Figure 12.**
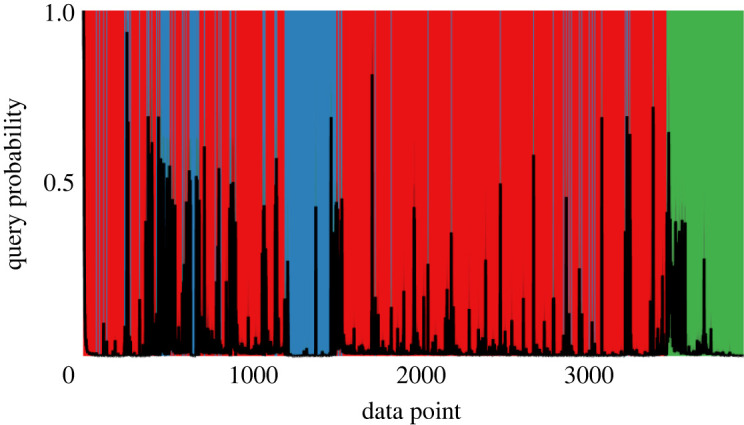
Query probability for the online (ordered) Z24 data. (Online version in colour.)

In the view of consistency (and to avoid parameter tuning) the hyperparameter values are consistent with the simulated example. To reiterate: the prior mean is the expected value of the data $\mu_{0}=E[x_{i}]$ and the prior covariance is based on the (expected) variance $\Sigma_{0}=(\nu +d+2)\times V[x_{i}]$; meanwhile, κ=1, ν=d, and α=10. For the ordered (online) data, the query probability is shown in figure 12, averaged over 100 repeats. The shaded background of the plot corresponds to the changing classes of data over time—the colour scheme is consistent with the supervised model in figure 11. Of interest is the reduction in query probability once the class corresponding to low temperature (blue) has been investigated—roughly from points 1250–1500. Since data have already been investigated in this class, the model can confidently return to classifying data in this regime without the need for further inspection, an important advantage over conventional novelty detection.

As with the simulated example, peaks in the query probability correspond to the introduction of new structural conditions or regions of the data stream that are mixed. As the classes have increased mixing (and reoccur) for the *in situ* bridge data, these patterns are more easily observed when comparing the query probability with changes in the shaded background of figure 12.

**Figure 13.**
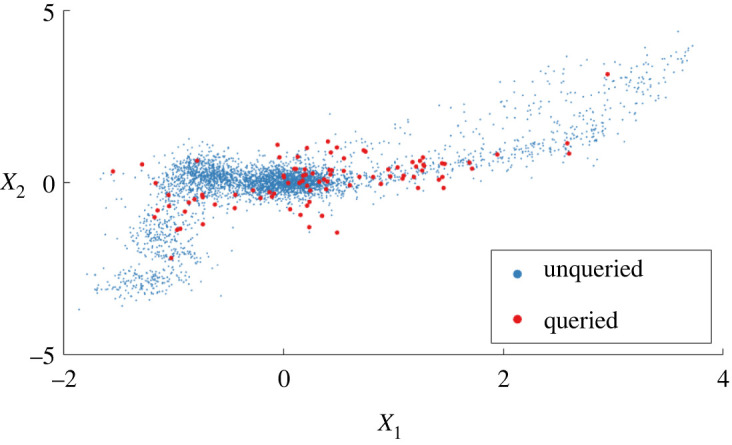
Active queries using information efficiency for online (ordered) Z24 data, plotting the first two features of the four-dimensional feature space. (Online version in colour.)

An example of active samples in the feature space is shown in figure 13 (for one trial, drawn at random). As expected, the leading edges of clusters and mixed regions are queried more frequently. Points close to the extremities of the data confirm that the model has identified an appropriate number of clusters. Paying particular attention to the point close to $X_{1}=3$, $X_{2}=3$, this investigation informs the model that a large variance component covers all of the low-temperature condition (blue class). On average, the DPGMM queries 117.9 data and achieves 95.32% classification accuracy. This corresponds to an automated inspection budget of just 3.00% for a performance accuracy of 95.32%, which compares with 97.20% for a fully supervised classifier (using 100% labelling).

The results for the *in-situ* bridge monitoring example reinforce the findings of the simulated example, §6. It is reassuring that improvements in performance—via semi-supervised learning—are also borne out in this full-scale example, confirming findings seen in [40]. In addition, the proposed active learning scheme, which is the novel contribution of this paper, appears to select an appropriate number of inspections given the SHM application (3%) and maintains performance close to a fully supervised model. The performance seen on this classic benchmark dataset [55] (in addition to the example data shown in §6) would suggest that the proposed methodology could be a powerful tool for assisting inspection management in SHM.

## 8. Concluding remarks

A new approach to managing inspections in SHM regimes has been introduced. The algorithm is important, as it negates the requirement for extensive data to train a model prior to system operation. Instead, the monitoring classifier is learnt incrementally from streaming data, recorded throughout the operational life. Most importantly, the algorithm automatically manages expert inspections by querying descriptions for specific (insightful) measurements from online data streams. In turn, the value of the monitoring regime is maximized, finding a trade-off between predictive performance and the cost of labelling.

A constrained Dirichlet process defines the semi-supervised model of measured data, which enables inferences from both labelled and unlabelled signals; while a novel active learning procedure is used to flag which measurements require inspection. As a result, inspection regimes can be automated (or enhanced) by the algorithm—which determines an appropriate inspection budget in a given application.

The approach is verified in a simulated SHM example and applied to an *in situ* bridge monitoring dataset. For the bridge monitoring data, an automated inspection regime reaches 98.07% of the fully supervised classification accuracy, while using just 3% of the labels, which would correspond to significant savings in practice.

## Data Availability

Data related to the Z24 benchmark study (§7) are publicly available, e.g. see https://bwk.kuleuven.be/bwm/z24. The model and data are available on GitHub GitHub.

## Footnotes

1 An established problem in practical applications of machine learning, often referred to as ‘high variance’ data [18].

2 Throughout this work, budget refers to the number of labels it is possible to obtain—the reason for this need not necessarily be financial.

3 It can also include methods of learning from weakly labelled data [24].

4 That is, the averaged Shannon information content [26] of the possible label outcomes k∈{1,…,K}, if there are K possible labels (i.e. performance or health states).

5 Note how this contrasts to active learning, whereby unlabelled instances are only sampled to extend the labelled training-set.

6 They are not necessarily consistent with the component index k in the DP. For instance, the engineer may label a datum as ‘Condition 2—high-speed operation’ but this does not imply an association with cluster k=2, indexed by the DP.

7 The notation of subscript −j, ${\left(\cdot \right)}_{-j}$, is adopted to denote all points except the jth.

8 This assumes the well-known *label switching problem* [24] can be resolved for the unsupervised case—in practice, this is often not possible.
